# Prognostic and onco-immunological value of immune-related eRNAs-driven genes in lung adenocarcinoma

**DOI:** 10.1007/s00432-024-05687-5

**Published:** 2024-04-11

**Authors:** Xuan Wu, Xingru Zhao, Chao Zhou, Nan Wei, Zhiwei Xu, Xiaoju Zhang

**Affiliations:** Department of Respiratory and Critical Care Medicine, Zhengzhou University People’s Hospital, Henan Provincial People’s Hospital, Weiwu Road No.7, Zhengzhou, 450003 Henan China

**Keywords:** Enhancer RNA, Immune, Prognosis, Lung adenocarcinoma, Biomarker

## Abstract

**Background:**

We aimed to comprehensively analyze the clinical value of immune-related eRNAs-driven genes in lung adenocarcinoma (LUAD) and find the potential biomarkers for prognosis and therapeutic response to improve the survival of this malignant disease.

**Materials and methods:**

Pearson’s correlation analysis was performed to identify the immune-related eRNAs-driven genes. Cox regression and least absolute shrinkage and selection operator (LASSO) analyses were used to construct this prognostic risk signature. Gene Ontology (GO) and Kyoto Encyclopedia of Genes and Genomes (KEGG) pathway analyses were used to investigate the underlying molecular mechanism. The single sample gene set enrichment analysis (ssGSEA) algorithm was conducted to evaluate the immune status based on the signature. The quantitative real-time PCR (qRT-PCR) analysis was performed to evaluate the expression value of the signature genes between LUAD tissues and adjacent lung tissues.

**Results:**

Five immune-related eRNAs-driven genes (*SHC1, GDF10, CCL14, FYN,* and *NOD1*) were identified to construct a prognostic risk signature with favorable predictive capacity. The patients with high-risk scores based on the signature were significantly associated with the malignant clinical features compared with those with low-risk scores. Kaplan–Meier analysis demonstrated that the sample in the low-risk group had a prolonged survival compared with those in the high-risk group. This risk signature was validated to have a promising predictive capacity and reliability in diverse clinical situations and independent cohorts. The functional enrichment analysis demonstrated that humoral immune response and intestinal immune network for IgA production pathway might be the underlying molecular mechanism related to the signature. The proportion of the vast majority of immune infiltrating cells in the high-risk group was significantly lower than that in the low-risk group, and the immunotherapy response rate in the low-risk group was significantly higher than that in the high-risk group. Moreover, BI-2536, sepantronium bromide, and ULK1 were the potential drugs for the treatment of patients with higher risk scores. Finally, the experiment in vivo and database analysis indicated that *CCL14, FYN, NOD1,* and *GDF10* are the potential LUAD suppressor and *SHC1* is a potential treatment target for LUAD.

**Conclusion:**

Above all, we constructed a prognostic risk signature with favorable predictive capacity in LUAD, which was significantly associated with malignant features, immunosuppressive tumor microenvironment, and immunotherapy response and may provide clinical benefit in clinical decisions.

**Supplementary Information:**

The online version contains supplementary material available at 10.1007/s00432-024-05687-5.

## Introduction

Lung cancer is the most prevalent malignancy in males and the second most prevalent malignancy in females after breast cancer. However, it has the highest mortality both in males and females. As we know, non-small cell lung cancer (NSCLC) remains the most commonly diagnosed pathological type of lung cancer and comprises two main types—lung squamous cell carcinoma (LUSC) and lung adenocarcinoma (LUAD) (Herbst et al. 2008; Siegel et al. 2020). It is reported that there were about 1.8 million newly diagnosed lung cancer cases and 1.6 million deaths, which accounts for about 13% of all the diagnosed cancer cases and 20% of all the cancer-related deaths, respectively. Because of the lack of clinical symptoms at the early stage, the patients with LUAD are often diagnosed at the advanced stage, which limits the treatment options and results in unfavorable survival outcomes (Hirsch et al. 2017). Despite the application of surgery, chemotherapy, targeted drugs and other means, the 5-year overall survival rate of patients diagnosed in late periods is still less than 20% (Hirsch et al. 2017). Therefore, it is necessary to find reliable and effective molecular biomarkers for early diagnosis and evaluation of the curative effect in LUAD.

With the progress of the high-throughput DNA sequencing technologies, non-coding RNA (ncRNA) may regulate gene expression and play a key regulatory role in shaping cellular activity, which makes them a new class of molecular targets for drug discovery (Anastasiadou et al. 2018; Slack and Chinnaiyan 2019; Wang et al. 2022). As a type of lncRNA transcribed from DNA enhancer regions, enhancer RNA (eRNA) acts as a biomarker for activated enhancers and plays essential roles in gene regulation. Accumulating evidence has showed that eRNAs are associated with multiple traits, characteristics, and cancers. For example, *NET1* eRNA regulates the expression of *neuroepithelial cell transforming 1 (NET1)* oncogene to promote tumorigenesis in breast cancer (Zhang et al. 2019) and *HPSE* eRNA regulates the expression of *heparinase (HPSE)* to promote the invasion and metastasis of cancer (Jiao et al. 2018). The eRNA disorders can affect biological processes, including cell cycle and cancer cell growth, or change the expression of target genes (Hsieh et al. 2014; Lam et al. 2013; Melo et al. 2013), adding new insights into the action mechanisms of enhancers. At the same time, some studies have found that the expression of some eRNA and their targeting genes are related to the clinical stage and prognosis of patients with LUAD and can be used as reliable biomarkers to predict immune status and therapeutic response (Cheng et al. 2021).

The eRNAs are considered to be an important regulatory layer of the epigenome and play an important role in almost all the biological processes, including immunity. The tumor microenvironment (TME) comprises immune and stromal cells, and the interactions between them and cancer cells play important roles in the initiation, progression, and response to therapies in lung cancer (Xiao and Yu 2021). The TME has been approved to be the potential target for anti-tumorigenesis treatment in cancers. The epigenetic alterations are always targeted for the development of molecular targets, which allows for generating novel therapeutic strategies to ultimately improve the survival outcomes of the patients suffering from this aggressive malignancy. The genetic, immune, and pharmacogenomic landscapes of eRNAs in LUAD remain unexplored.

Here, we generated a co-expression network to identify the eRNAs of target immune-related genes to comprehensively analyze the role in the prognosis and therapeutic response of LUAD patients. Moreover, we constructed a prognostic risk signature with five immune-related eRNAs-driven genes by bioinformatics methods and further verified its reliability and sensitivity.

## Materials and methods

### Data acquisition and preprocessing

The gene sequence, profile, and related clinical data of LUAD patients were acquired from The Cancer Genome Atlas (TCGA) public database. The samples with missing clinical information and/or overall survival ≤ 30 days were excluded from further analysis. Finally, 492 samples were included in this study. The information about clinical samples, for example, age, sex, and the tumor stage are shown in Supplemental Table 1. The expression data of eRNAs and the target genes were obtained from a previous study (Zhang et al. 2019). The immune-related genes were acquired from ImmPort Portal. A total of 562 eRNAs and 1652 target genes were identified in LUAD (Spearman's correlation coefficients > 0.3). Of these 1,652 target genes, 114 are immune related. The eRNAs and target immune-related genes are listed in Supplementary Table 2. All eRNAs identified and their regulatory relationship with the target genes are shown in Supplementary Table 3.

### Functional enrichment analysis

In this study, Gene Ontology (GO) term and Kyoto Encyclopedia of Genes and Genomes (KEGG) pathway analyses were conducted to investigate the potential molecular mechanism involved in the 114 immune-related eRNA-driven genes for LUAD using the “clusterProfiler” R package.

### Immune-related eRNA-driven gene prognostic signature

To construct a reliable prognostic risk signature, the LUAD samples were randomly assigned to the training (*n* = 482) and the testing cohorts (*n* = 482) in a ratio of 1:1. Then, least absolute shrinkage and selection operator (LASSO) analysis was conducted on the 114 immune-related eRNA-driven genes to restrict overfitting among these genes significantly related to each other using the “glmnet” R package in the training cohort. Finally, five genes were identified to construct the prognostic risk signature based on the expression value of each signature gene and the relevant coefficient. The formula is as follows. Risk score = 0.208 × *SHC1* + (−0.040) × *CCL14* + (−0.061) × *FYN* + (−0.075) × *NOD1* + (−0.008) × *GDF10*.

### Validation of this immune-related eRNA-driven gene signature

The risk score of each sample was calculated and the samples were then assigned into the high-risk group and the low-risk group by the median risk value. Moreover, Kaplan–Meier curve analysis with the log-rank test was implemented to evaluate the correlation between the risk score and the survival of LUAD patients through the “survival” and “surviminer” R packages. Receiver operating characteristic (ROC) curves as well as the area under the curve (AUC) values were performed to evaluate the performance of the signature in predicting the survival rate of the LUAD patients. We also validated the predictive capacity in two independent GEO cohorts (GSE50081, *n* = 181 and GSE68465, *n* = 462).

### Sample collection

We collected the samples of tumor and adjacent non-tumor tissue from three LUAD patients who underwent surgery at Zhengzhou University People’s Hospital. All patients included in this study gave permission for sampling by signing a written informed consent form. This study was approved by the ethical review board of Zhengzhou University People's Hospital.

### RNA extraction and real-time reverse transcriptase–polymerase chain reaction

We extracted total RNA from the sample tissues using TRIzol based on the manufacturer’s instructions. Then, cDNA was synthesized using the PrimeScript TMRT kit (Takara, Japan). Real-time polymerase chain reaction (RT-PCR) was conducted using SYBR Green Master Mix (Yeasen, China). The expression of each mRNA was standardized to the level of mRNA *actin*, and the quantification of expression was executed using the 2–ΔΔCT method. The sequence of each primer used in this study was summarized as follows.

*Actin*-F, 5ʹ-TGGCACCCAGCACAATGAA-3ʹ,

*Actin*-R, 5ʹ-CTAAGTCATAGTCCGCCTAGAAGCA-3ʹ,

*CCL14*-F, 5ʹ- GCCATTCCCTTCTTCCTCCT-3ʹ,

*CCL14*-R, 5ʹ- GACGCGGGATCTTGTAGGTA-3ʹ,

*FYN*-F, 5ʹ-GGTGTGAACTCTTCGTCTCATA-3ʹ,

*FYN*-R, 5ʹ-TGTCCGTGCTTCATAGTCATAA-3ʹ,

*GDF10*-F, 5ʹ-CGGCTGGAATGAATGGATAATC-3ʹ,

*GDF10*-R, 5ʹ-TTGGATGGACGAACGATCTTAG-3ʹ,

*NOD1*-F, 5ʹ-GTCCGAGTTCTTCCTCTACTTG-3ʹ,

*NOD1*-R, 5ʹ-CCATGATGGTGTCCATGTAGAT-3ʹ,

*SHC1*-F, 5ʹ-ACTTGGGAGCTACATTGCCT-3ʹ,

*SHC1*-R, 5ʹ-GGGTGCACTGCCATTGATAG-3ʹ.

### Construction and evaluation of the nomogram

To estimate the clinical application of this prognostic risk signature in LUAD, a nomogram was constructed with the risk score and classical clinical variables, including age, gender, tumor stage, and TNM stage, using “rms” R package. Time-dependent ROCs and the AUCs were implemented to evaluate the performance of the nomogram in predicting the survival rates at 1, 2, and 3 years through “ROCsurvival” R package.

### Immune cell infiltration and immunotherapy response related to the signature

Immune-infiltrating cells in TME play important roles in cancer progression and therapeutic response. The hub gene set that consists of 782 genes representing 28 types of immune cells is used to estimate the infiltration level of various immune cell types in TME. Then, the single sample gene set enrichment analysis (ssGSEA) algorithm was executed to evaluate the proportion of 28 types of immune cells based on the gene expression profiles. The immunologic features between the two risk groups were evaluated by the ssGSEA algorithm using the “GSVA” R package. In addition, the immune/stromal/estimate scores and tumor purity in TME were calculated based on the transcriptome data by ESTIMATE algorithm using the “estimate” R package. Tumor immune dysfunction and rejection (TIDE) score was determined to evaluate the potential immune checkpoint inhibitor (ICI) response. The lower the TIDE score, the better is the response to immunotherapy. The potential ICI responses between the two risk groups were evaluated using the “ggpubr” R package.

### Drug sensitivity analysis

The clinical drug responses in LUAD patients between the two risk groups were evaluated based on the half-maximal inhibitory concentration (IC50) of different anti-cancer drugs. The anti-cancer drug datasets were acquired from Drug Sensitivity in Cancer (GDSC) website to evaluate the correlation between the IC50 values of different anti-cancer drugs and the risk scores using “oncoPredict” R package (Maeser et al. 2021). The clinical responses to these drugs between the two risk groups were also explored to provide novel insights into the precision treatment of LUAD patients.

### Statistical analysis

Pearson’s correlation analysis was used to identify the eRNAs-driven genes. Univariate and multivariate analyses were carried out to identify the prognostic factors in LUAD. The survival status was evaluated by Kaplan–Meier curve analysis with log-rank tests. ROCs analysis and the AUCs were carried out to assess the reliability and sensitivity of the prognostic risk signature. Student’s *t* test was used to evaluate the difference between the two groups, and *P* < 0.05 was considered to be significant difference.

## Results

### Immune-related eRNA-driven gene prognostic signature for LUAD patients

**Fig. 1:**
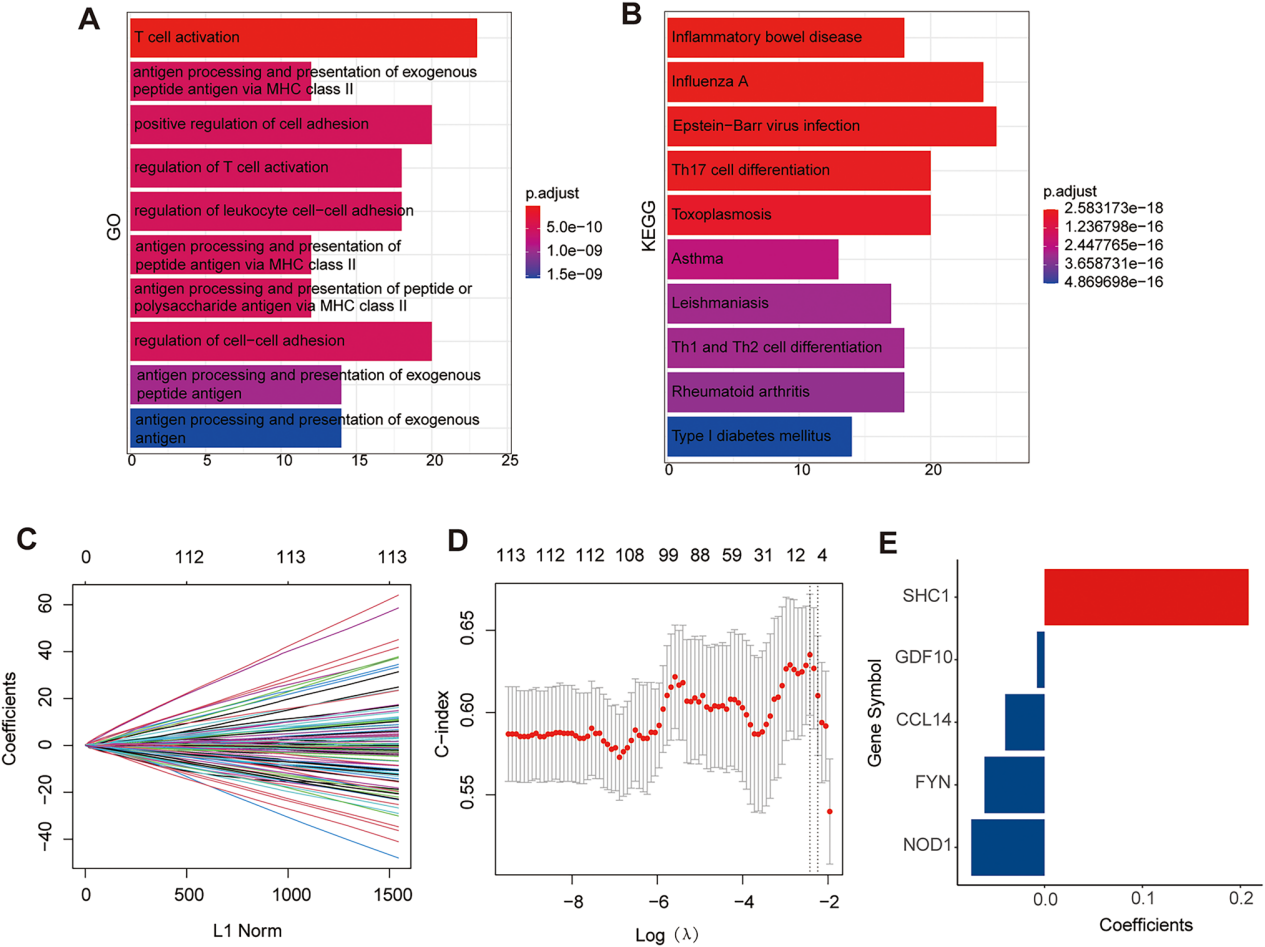
The identification of the five immune-related eRNA-driven genes in the signature. **A** The Gene Ontology (GO) biological processes associated with these immune-related eRNA-driven genes. **B** Kyoto Encyclopedia of Genes and Genomes (KEGG) pathways associated with these immune-related eRNA-driven genes. **C** Adjusting the parameters of overall survival (OS)-related genes to validate the error curve. **D** The minimum criteria can be determined by using imaginary lines that intersect perpendicularly. **E** The five immune-related eRNA-driven genes and the relevant coefficients

We identified 1202 eRNAs in LUAD, which regulate 1716 target genes from the TCGA database. Based on the criterion of Pearson correlation coefficient > 0.3 and *p* < 0.05, 562 eRNAs and 1652 target genes were acquired, including 114 immune-related genes in LUAD (Supplemental Table 2). GO and KEGG analyses showed that a number of important immune processes were involved in these 114 genes, such as T cell activation, antigen processing and presentation of exogenous peptide antigen via MHC class II and positive regulation of cell adhesion, and Th17 cell differentiation, which indicate the significant correlation between the immune-related eRNA-driven genes and the immune status (Fig. 1A and B).

To develop a reliable prognostic risk signature, the LUAD samples in TCGA were randomly assigned to the training and testing cohorts. The immune-related eRNA-driven genes significantly associated with survival time were identified with the log-rank test and subsequently analyzed by LASSO regression model (Fig. 1C and D). Finally, five immune-related eRNA-driven genes (*SHC1, GDF10, CCL14, FYN,* and *NOD1*) were identified to construct this risk signature (Fig. 1E).

### Predictive performance of the signature

**Fig. 2:**
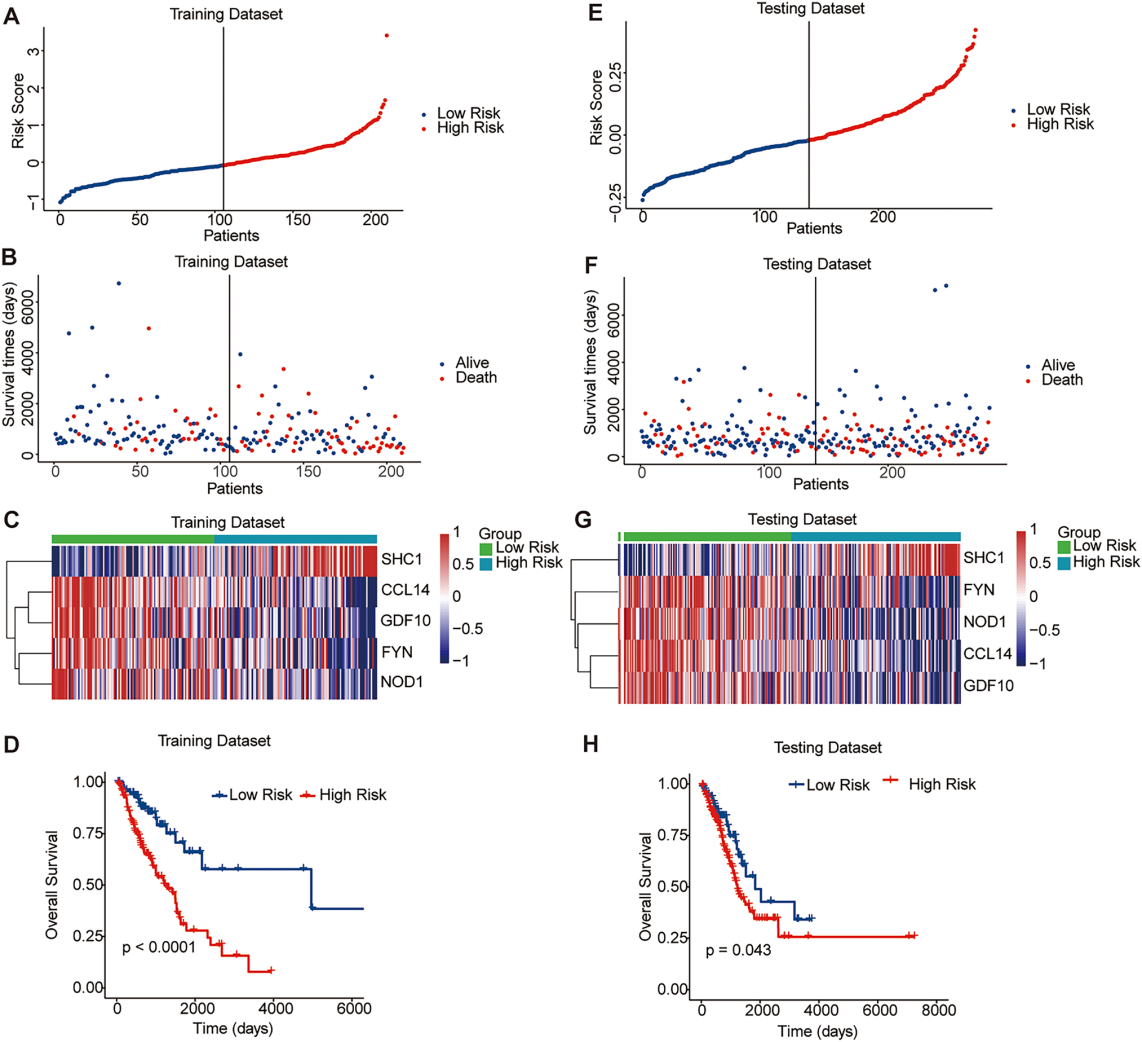
The correlation between the prognostic risk signature and the survival. The distribution plot **A**, scatter plot **B**, and expression heatmap of the five immune-related eRNA-driven genes **C** and Kaplan–Meier curve **D** between the high- and low-risk groups in the training cohort. The distribution plot **E**, scatter plot **F**, expression heatmap of the five immune-related eRNA-driven genes **G**, and Kaplan–Meier curve **H** between the high- and low-risk groups in the testing cohort

To validate the predictive performance of the prognostic risk signature, the LUAD samples in the training cohort were then assigned to the high-risk group and the low-risk group according to the median risk value. The distribution plot and scatter plot revealed that the high-risk group had an increased mortality (Fig. 2A and B). Moreover, the heatmap indicated that the *SHC1* expression was increased, whereas *CCL14, FYN, NOD1* and *GDF10* expressions were decreased in the high-risk group (Fig. 2C). Besides, Kaplan–Meier analysis demonstrated that the sample in the low-risk group had a prolonged survival compared with those in the high-risk group (Fig. 2D).

To further prove the predictive performance of this risk signature, we also calculated the risk score of each sample in the testing cohort based on the same formula and similarly assigned to the high- and low-risk groups according to the median risk score. The results of the distribution plot, scatter plot, heatmap analyses, and Kaplan–Meier curve in the testing cohort were consistent with the results acquired from the training cohort (Fig. 2E-H). In summary, the high-risk score of the patients calculated by the signature formula indicated unfavorable survival outcomes in LUAD accurately.

### Validation in two Independent GEO cohorts

**Fig. 3:**
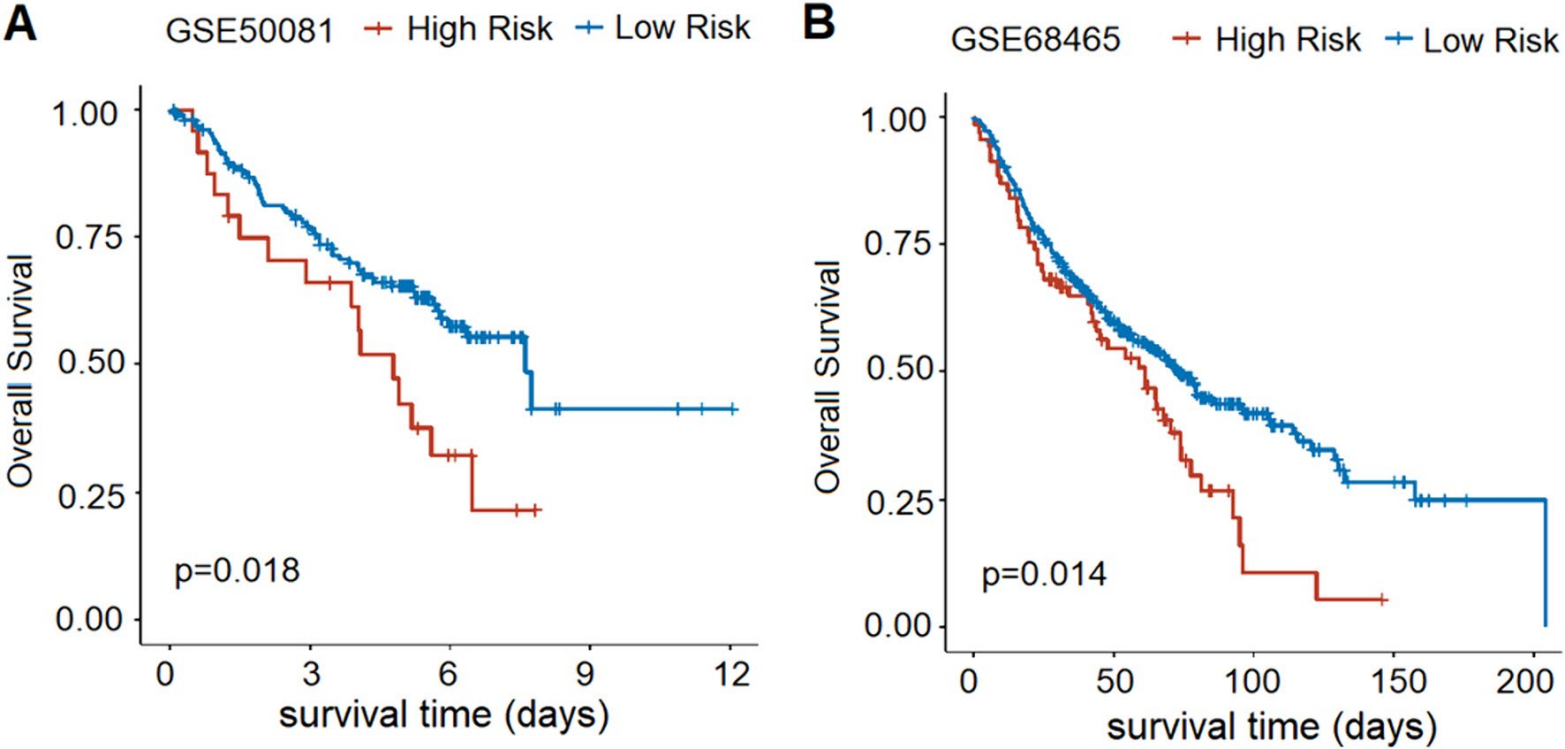
Prognostic value validation of the risk signature in three independent GEO cohorts. **A** Survival status between the high-risk group and the low-risk group in the GSE50081 cohort. **B** Survival status between the high-risk group and the low-risk group in the GSE68465 cohort

In addition, we validated the capacity of this signature in two independent cohorts (GSE50081 and GSE68465) from the GEO database (Fig. 3). Kaplan–Meier survival curves demonstrated that the low-risk patients had significantly prolonged survival compared with the high-risk patients, which further validated the reliability of the signature.

**Fig. 4:**
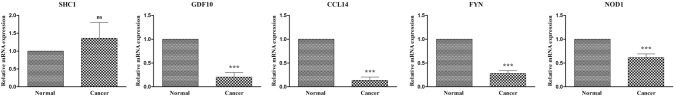
The mRNA expressions between tumor and adjacent non-tumor tissues of five signature genes, including *SHC1* **A**, *GDF10* **B**, *CCL14* **C**, *FYN* (D), and *NOD1* (E). *P < 0.05, **P < 0.01, and ***P < 0.001

We further evaluated the mRNA expression of five signature genes between three-paired tumor tissues and adjacent non-tumor tissues. As shown in Fig. 4, *CCL14, FYN, NOD1,* and *GDF10* mRNA expressions were decreased in tumor tissues compared with the adjacent non-tumor tissues. Besides, the *SHC1* mRNA expression tends to be increased in tumor tissues compared with the adjacent non-tumor tissues. PCR results together with database analysis revealed that *CCL14, FYN, NOD1,* and *GDF10* are the potential LUAD suppressors and *SHC1* is a potential treatment target for LUAD.

### Correlation between clinicopathological features and risk score

**Fig. 5:**
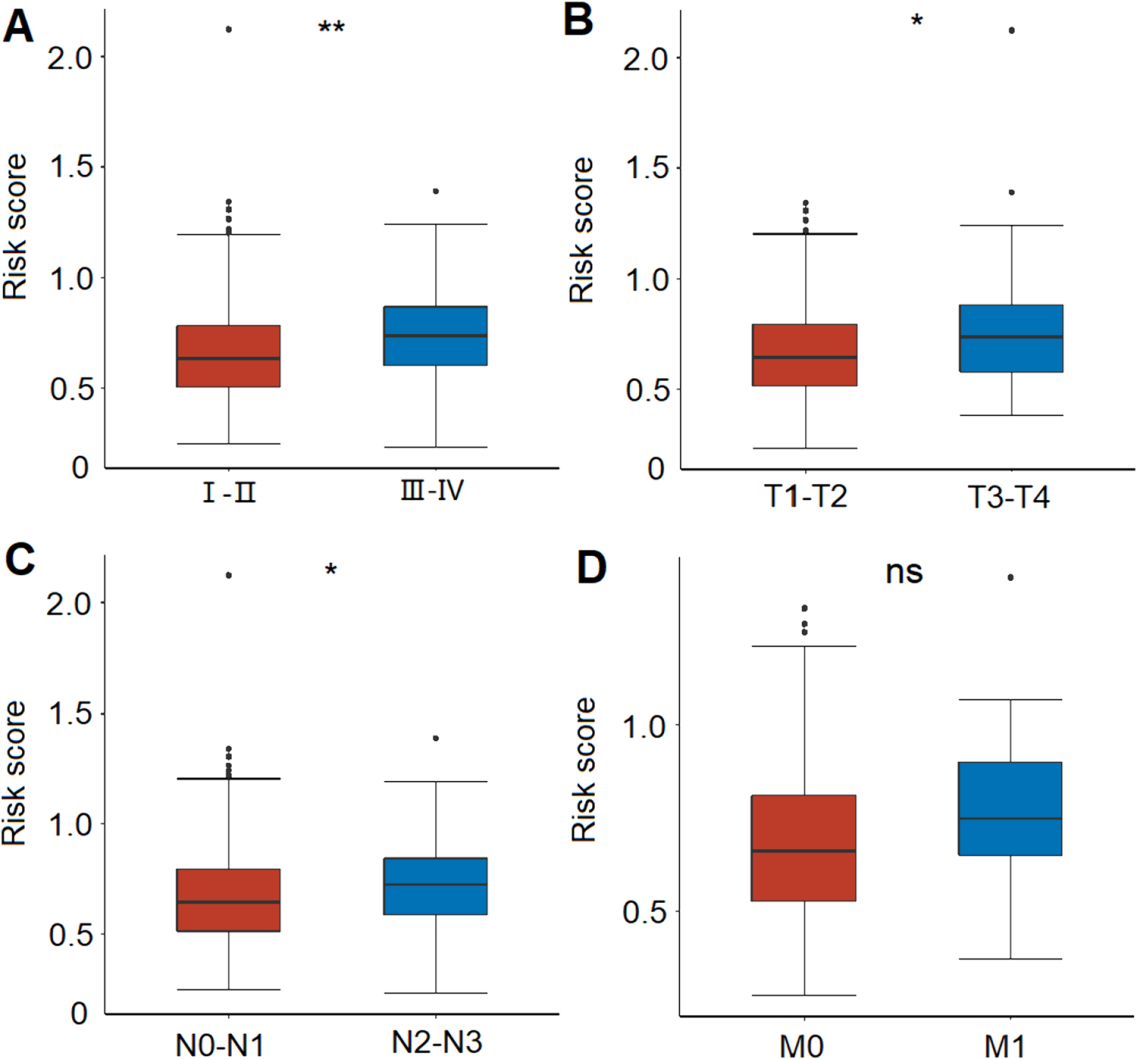
The correlation between the risk score and clinicopathological features. **A** The correlation between the risk score and tumor stage (I–II and III–IV). **B** The correlation between the risk score and T stage (T1–T2 and T3–T4). **C** The correlation between the risk score and N stage (N0–N1 and N2–N3). **D** The correlation between the risk score and M stage (M0 and M1). *P < 0.05, **P < 0.01

**Fig. 6:**
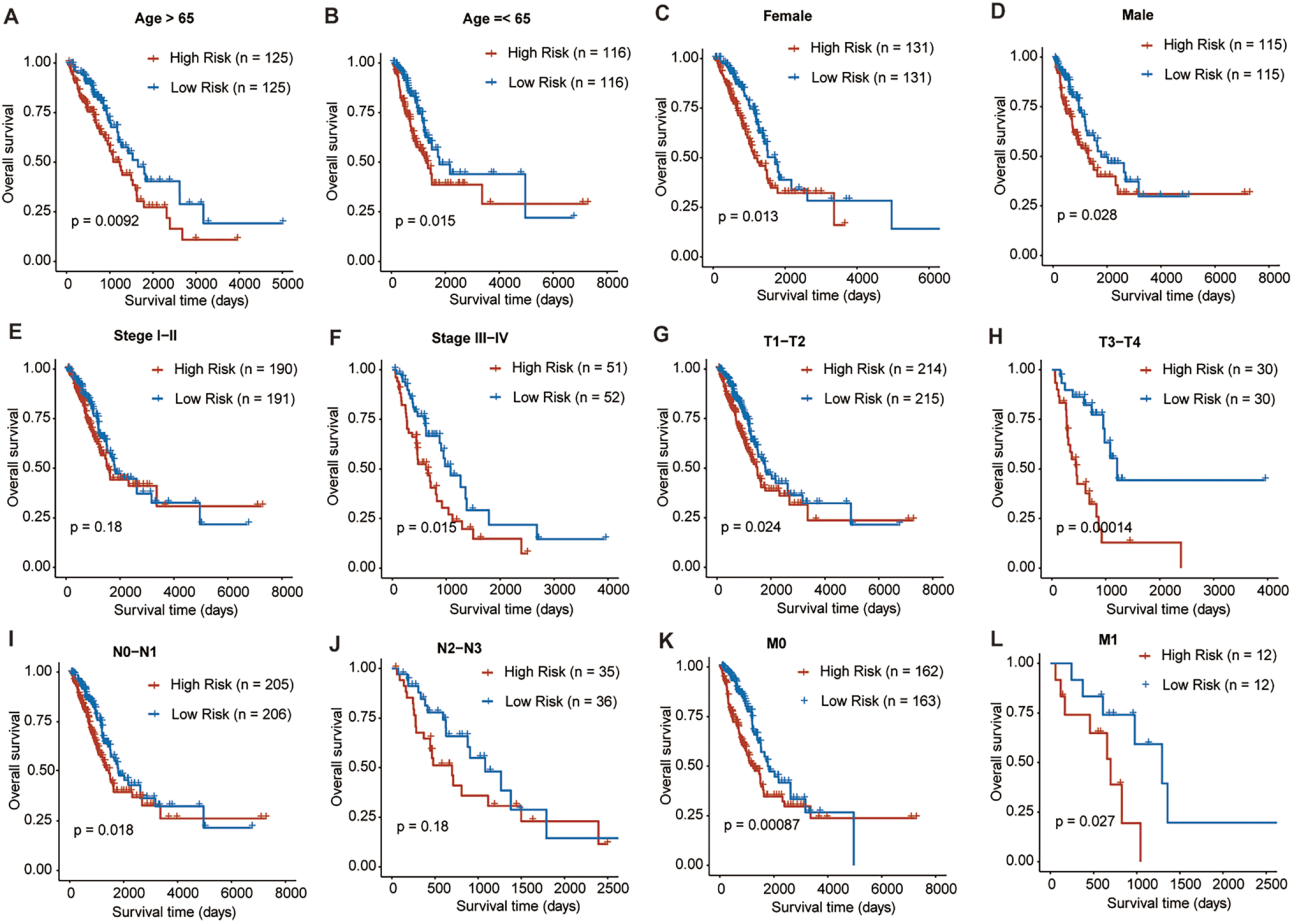
The survival status between the two risk groups in different subgroups of patients with LUAD. The survival outcomes of the LUAD patients stratified according to age **A**, **B** gender **C**, **D** stage **E**, **F** T stage **G**, **H** N stage **I**, **J** and M stage **K**, **L** between the two risk groups

We also evaluated the correlation between the risk score and clinicopathological features, such as the age (< 65 and ≥ 65 years), gender (female and male), T stage (T1–T2 and T3–T4), N stage (N0–N1 and N2–N3), M stage (M0 and M1), and tumor stage (I–II and III–IV). The high risk scores were significantly associated with the malignant features, including advanced stage (III–IV), larger tumors (T3-T4), more lymph node metastases (N2–N3), and distant metastasis (M1) (Fig. 5). Kaplan–Meier analysis demonstrated that the patients in the high-risk group had worse survival compared with those in the low-risk group in the most cohorts stratified by the clinicopathological features, except for advanced stage (I-II), and N2–N3 stage cohorts (Fig. 6). The results showed that this risk signature had the promising predictive capacity in diverse situations.

### Independent prognostic and clinicopathological correlation analyses

**Fig. 7:**
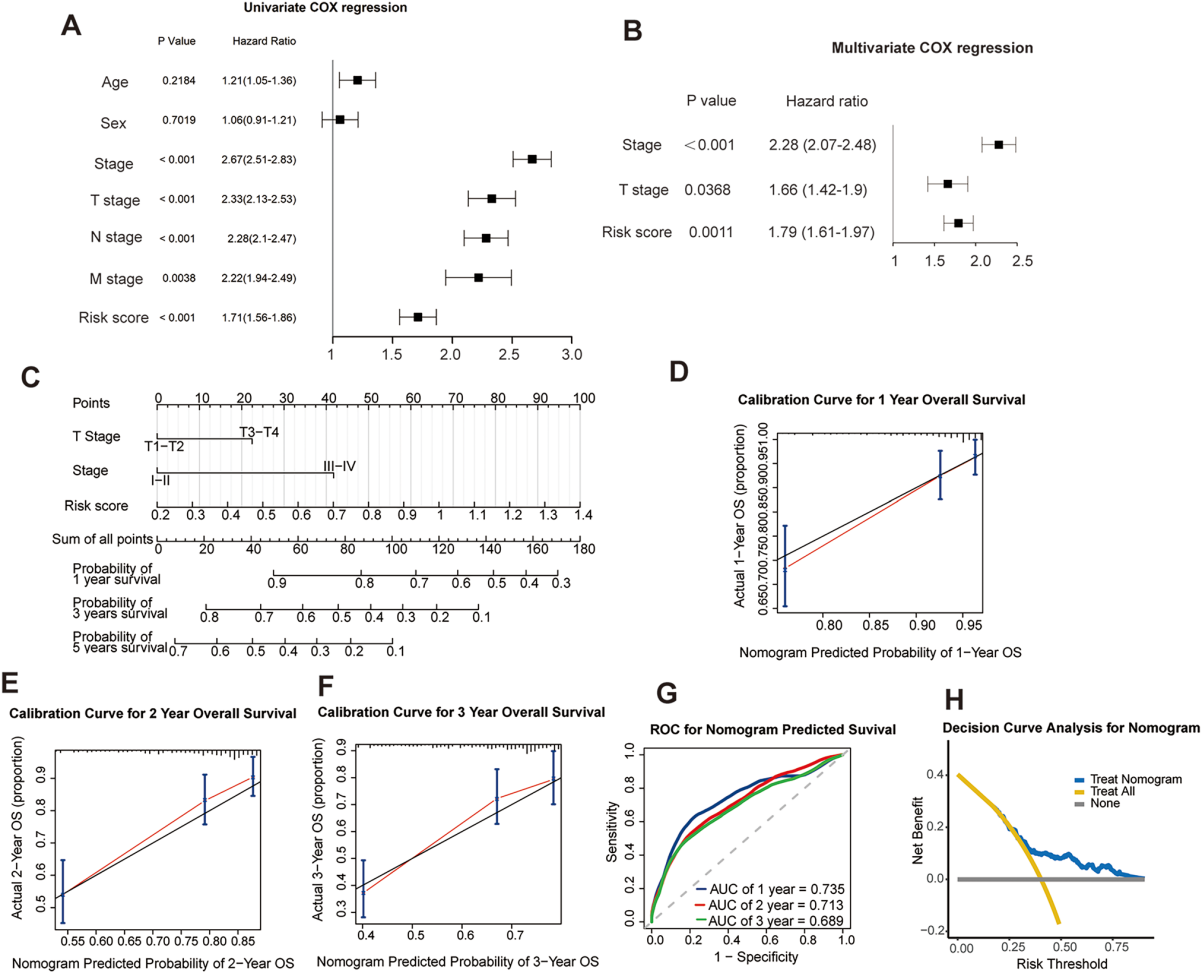
Construction and validation of the nomogram based on the clinicopathological features and risk score. **A**, **B** The independent prognostic factors identified by univariate and multivariate Cox regression analyses. **C** Construction of the prognostic nomogram based on the clinicopathological features and risk score. **D**-**F** Calibration curves between the predictive survival probability and actual survival rates at 1 year, 2 years, and 3 years. **G** Time-dependent receiver operating characteristic curves of the nomogram and the area under the curve values at 1, 2, and 3 years. **H** Decision curve analysis of the prognostic nomogram

We further explored the independent prognostic value of this signature as well as the common clinicopathological features using univariate and multivariate analyses. The univariate Cox regression analysis showed that the risk score as well as tumor stage, and TNM stage, correlated significantly with poor survival (Fig. 7A). The subsequent multivariate Cox regression analysis indicated that the risk score, tumor stage, and T stage were independent prognostic factors of LUAD patients (Fig. 7B). Then, a prognostic nomogram was constructed with the risk score and these clinicopathological features to quantitatively estimate the survival probability of LUAD patients (Fig. 7C). Moreover, calibration curves for 1-year, 2-year, and 3-year overall survival were performed to validate the predictive probability of this nomogram. The result indicated excellent concordance between the predictive survival probability and actual survival rates at 1 year, 2 years, and 3 years (Fig. 7D-F). Moreover, time-dependent ROCs exhibited that the nomogram had a promising accuracy in predicting the 1-, 2- and 3-year overall survival (AUC = 0.74, 0.71, and 0.69, respectively) (Fig. 7G). Furthermore, decision curve analysis was used to evaluate whether this prediction signature for treatment benefit would lead to better clinical decisions. The result showed the use of the nomogram in predicting the survival of LUAD patients brought more net benefit than treating either all or none at a range of clinically reasonable risk thresholds (Fig. 7H). All the above results showed that this prognostic risk signature displayed favorable predictive performance in the survival of LUAD patients and had a potential value of clinical applications.

### Functional enrichment analysis related to the signature

**Fig. 8:**
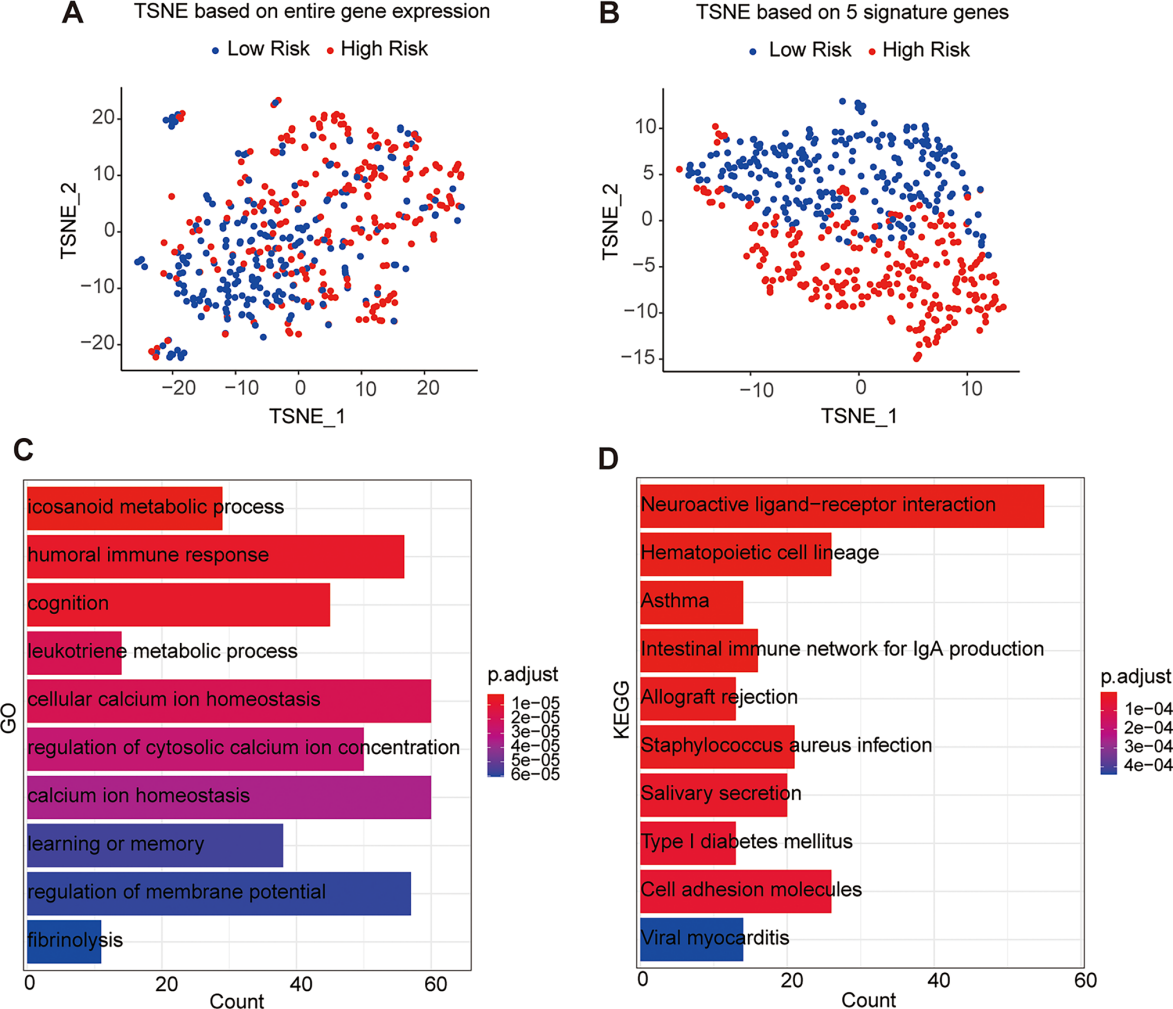
Functional enrichment analysis related to the risk signature. **A** t-SNE based on the entire gene expression. **B** t-SNE based on the signature gene expression. **C** Gene Ontology (GO) biologic processes in which DEGs are mainly enriched. **D** Kyoto Encyclopedia of Genes and Genomes (KEGG) pathways in which DEGs are mainly enriched

We performed t-distributed stochastic neighborhood embedding (t-SNE) to make a distinction between the two risk groups. The t-SNE analysis based on the signature genes showed that the two risk groups had different distributions and could be easily distinguished (Fig. 8A and B).

Then, GO and KEGG analyses were performed to explore the potential molecular mechanisms underlying the differentially expressed genes (DEGs) between the two risk groups defined by |log2 (fold change) > 1| and *p* < 0.05. The results showed that the humoral immune response was mainly enriched (Fig. 8C). For KEGG items, the intestinal immune network for IgA production pathway was mainly enriched (Fig. 8D).

### Correlation between immune and risk score

**Fig. 9:**
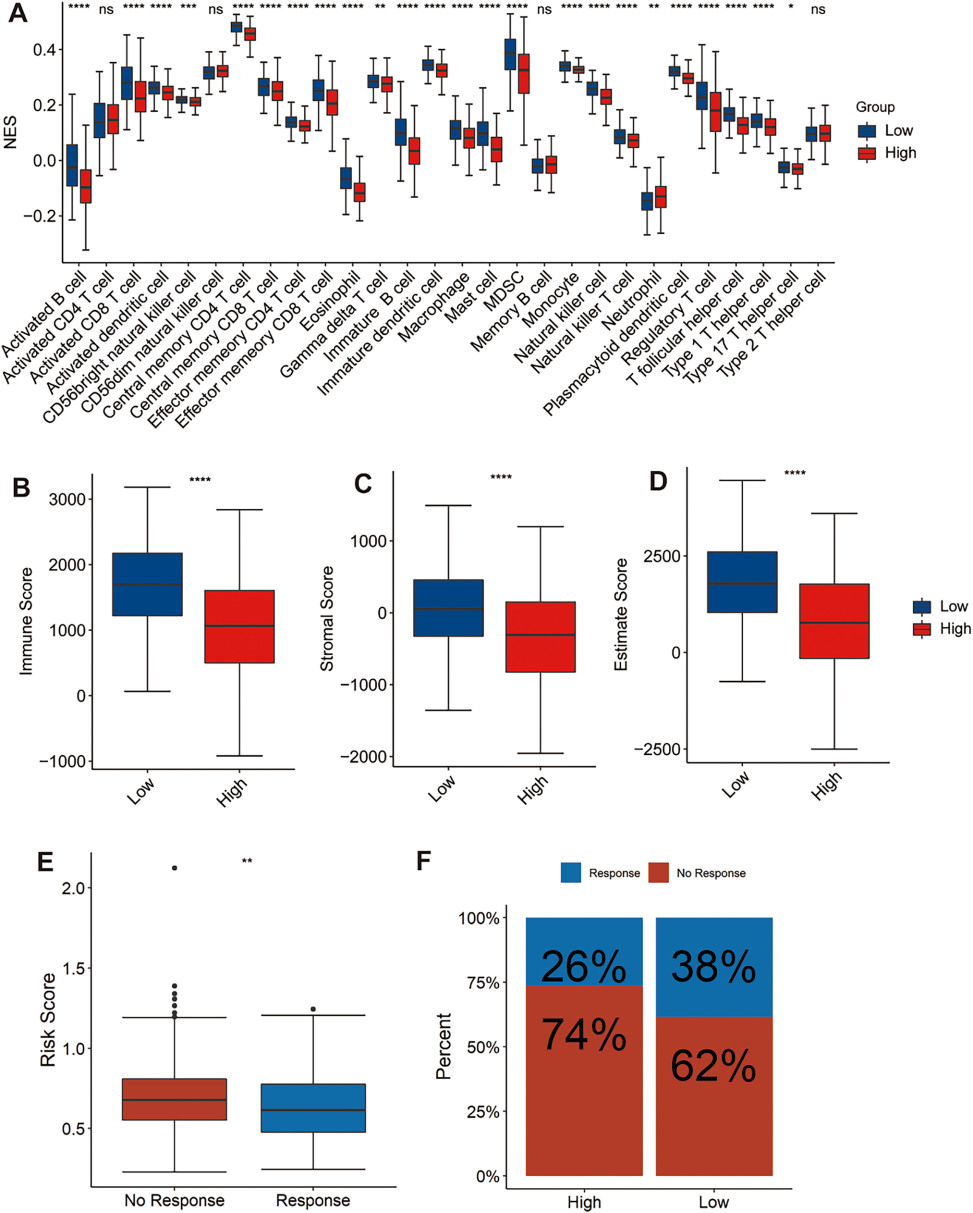
The correlation between the risk signature and immune responses. **A** The relative quantity of each immune-infiltrating cell type between the two risk groups. **B**, **C** and **D** The immune/stromal/ESTIMATE score between the two risk groups. **E** The immunotherapy responses between the two risk groups. **F** The risk scores between the no-response and response groups. *P < 0.05, **P < 0.01, ***P < 0.001, ****P < 0.0001

To further explore the clinical value of the prognostic risk signature in immunotherapy, we evaluated the relationship between immune cell infiltration and this signature. The results showed that the proportion of the vast majority of immune-infiltrating cells in the high-risk group was significantly lower than that in the low-risk group, such as activated B cell, immature B cell, CD8 T cell, effector memory CD8 T cell, dendritic cell, central memory CD4 T cell, eosinophil, immature dendritic cell, macrophage, myeloid-derived suppressor cells (MDSCs), natural killer T cell, natural killer cell, regulatory T cell, type 1 T helper cell, and type 17 T helper cell (Fig. 9A).

The correlation between the risk signature and immune/stromal/ESTIMATE score was evaluated through estimate algorithm. The results revealed that immune/stromal/ESTIMATE scores in the high-risk group were significantly lower than those in the low-risk group (Fig. 9B-D).

Moreover, we also explored the correlation between the immunotherapy response rate and the risk signature based on the TIDE score. The analysis indicated that the low-risk scores were significantly related to a higher response rate than the high-risk scores, and the risk scores in the response cohort were significantly high compared with those in the response cohort (Fig. 9E-F). Above all, this risk signature in our study may be highly related to the immunosuppressive TME and may be a potential biomarker of immunotherapy response in LUAD.

### Drug sensitivity prediction

**Fig. 10:**
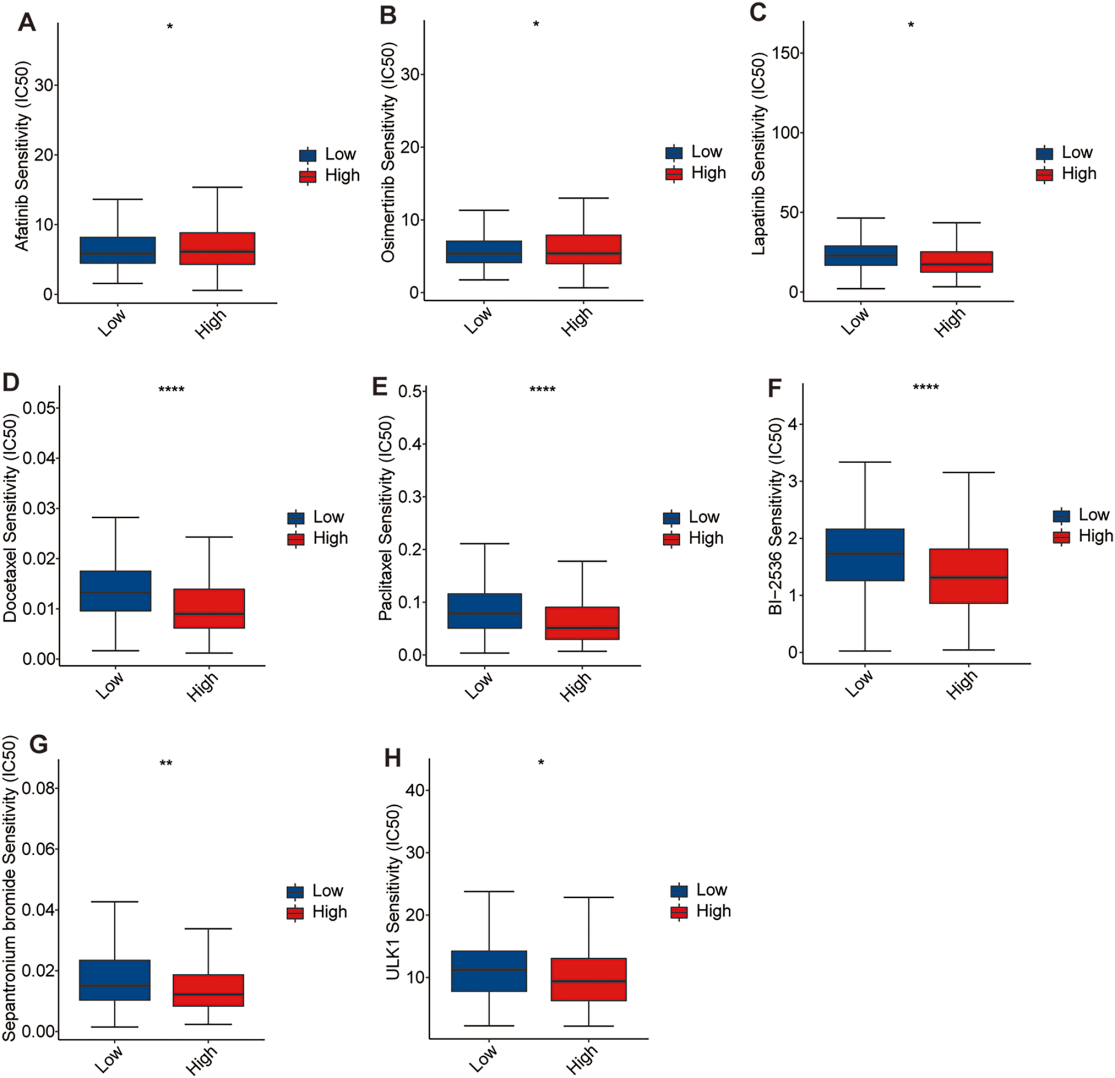
The correlation between the risk scores and sensitivities of anti-cancer drugs. Anti-tumor drugs, including **A** afatinib, **B** osimertinib, **C** lapatinib, **D** docetaxel, **E** paclitaxel, **F** BI-2536, **G** sepantronium bromide, and (H) ULK1. *P < 0.05, **P < 0.01, ****P < 0.0001

We evaluated the correlation between the risk scores and sensitivities of common anti-cancer drugs based on the GDSC database using “oncoPredict” R package. The result showed that the IC50s of lapatinib, docetaxel, and paclitaxel were lower in patients with high-risk scores; however, increased risk scores were accompanied by decreased sensitivity to afatinib and osimertinib (Fig. 10A-E). In addition, we also found that the IC50s of BI-2536, sepantronium bromide, and ULK1 were significantly higher in the low-risk group than those in the high-risk group, which suggests that BI-2536, sepantronium bromide, and ULK1 were also the potential drugs for the treatment of patients with higher risk scores (Fig. 10F–H).

## Discussion

TME is an integral part of LUAD and rich in inflammatory signaling, which attracts various immune cell populations (Allavena et al. 2011; Pitt et al. 2016; Spella and Stathopoulos 2021). Among them, tumor-reactive lymphocytes, tumor-associated macrophages (TAMs), tumor-associated neutrophils, myeloid-derived suppressor cells, and mast cells can interact with the tumor cells to ultimately shape a highly immunosuppressive TME, with enhanced tumor-promoting manifestations and diminished tumor cytotoxicity (Bronte et al. 2006; Ostrand-Rosenberg et al. 2012; Zaynagetdinov et al. 2011). Meanwhile, the efficacy of cancer immunotherapy in NSCLC is hampered by intra-tumor heterogeneity for neoantigens, neoantigen loss, immunosuppressive TME secondary to tumor aneuploidy, and immune checkpoints such as PD-L1 and TIM-3 (Anichini et al. 2018; Anichini et al. 2020; Koyama et al. 2016; Rosenthal et al. 2019). The transformation of immune response from the anti-tumor state to the tumor tolerance state is conducive to the development and progress of LUAD. Some molecular subsets show specific mechanisms for promoting immunotherapy resistance due to genetic alterations in NSCLC (Dong et al. 2017). Immune-related genes and immune cells are considered as new therapeutic targets and prognostic biomarkers of LUAD.

The eRNAs regulate gene expression and are involved in cancer immunotherapy by providing additional explanatory power in predicting immune response (Chen and Liang 2020). The eRNAs regulate gene expression in two ways: one is that the early formed eRNAs can recruit protein complexes from their synthetic site for local activation; the other is that eRNAs can recruit remote or even other chromosome-related protein complexes to play a distal regulatory role (Chen and Liang 2020; Wang et al. 2011). Accumulating evidence showed that the eRNAs are important regulators in the immune response and associated with multiple tumorigenic signaling pathways, including immune checkpoints, p53, and PPARr (Guo et al. 2020; Melo et al. 2013; Zhang et al. 2019).

In our study, we identified five immune-related eRNA-driven genes (*SHC1, CCL14, FYN, NOD1,* and *GDF10*) to construct a new prognostic risk signature and validated its predictive capacity. Of the five genes, *SHC1* expression correlated positively with poor survival. In contrast, *CCL14, FYN, NOD1,* and *GDF10* acted as protective factors (Fig. 1E and Supplementary Fig. 1).

Furthermore, the risk signature had promising predictive capacity in diverse situations. The high-risk patients were significantly associated with the malignant features, including increased mortality, advanced stage, larger tumors, more lymph node metastases, distant metastasis, and poor survival outcome. Moreover, we also validated the capacity of this signature in two independent cohorts from the GEO database.

Among the signature genes identified in this study, *SHC1* expression was significantly increased in patients with lung cancer, and its expression level and methylation level were associated with survival (Liang et al. 2021). In addition, *SHC1* was also significantly associated with DNA methylation, m6A RNA methylation, tumor mutational burden (TMB), Mismatch repair proteins (MMRs), microsatellite instability (MSI), TAMs, tumor-associated immune cell infiltration, and immune checkpoints in cancers (Chen et al. 2022). Pan et al. showed that *SHC1* was overexpressed in LUAD and interacted with *EGFR* to promote the metastasis of lung cancer cells. The complex of *SHC1* and *EGFR* was the potential therapeutic target to restrain lung cancer metastasis (Yang et al. 2022).

*CCL14* was considered to be a good prognostic biomarker in multiple cancer types and triggers the activation of monocytes, macrophages, and THP-1 cells through its binding affinity with *CCR1, CCR3,* and *CCR5*. Multiple studies suggest that *CCL14* contributes to the development and advancement of different medical conditions, such as allergic airway inflammation and certain types of cancer (Gu et al. 2020; Wong et al. 2016; Zhu et al. 2019). However, the roles of *CCL14* have not been described in LUAD.

As a TGFβ family member, *GDF10* is highly expressed in the lung. Overexpressing *GDF10* could attenuate tumor formation. Conversely, *GDF10* expression silence reversed these effects (Upadhyay et al. 2011), and *GDF10* is regarded as a tumor growth inhibitor and a silenced gene in lung cancers (Chen et al. 2023; Dai et al. 2004; Tandon et al. 2012).

As a non-receptor tyrosine kinase in the Src family of kinases, *FYN* plays important roles in the epithelial–mesenchymal transition (EMT) through regulating cell proliferation, morphology, apoptosis, and motor ability, which promotes tumorigenesis and progression, and is significantly associated with patients’ prognosis (Goel and Lukong 2016). *FYN* tyrosine kinase is a downstream target of receptor tyrosine kinases and modulates the immunotherapy response in the glioma (Comba et al. 2020). However, the role of *FYN* in LUAD has not been described.

Zhang et al. observed that *NOD1* and *NOD2* overexpression promote tumorigenicity and metastasis through the NOD1/2-NF-κb/ERK and IL-8 axis in human squamous cervical cancer (Zhang et al. 2022). However, Nod1 was also found to be an innate immune receptor and protects the intestine from inflammation-induced tumorigenesis (Chen et al. 2008). *NOD1* absence was associated with tumor growth and cell proliferation induced by an increased sensitivity to estrogen in MCF-7 cells (Silva et al. 2006). However, the role of *NOD1* required further study in LUAD.

We utilized GO and KEGG analyses to investigate the underlying molecular mechanism of the prognostic risk signature. Our findings indicate that this gene signature may play a critical role in regulating these tumor-related immune pathways. Additionally, recent research has shown that many types of LUAD are immunogenic and sponged in cancer-infiltrating lymph cells (Steven et al. 2016). Our team examined the relationship between the signature and immune infiltration in LUAD using the ssGSEA algorithm. Interestingly, we found that the risk signature was significantly associated with the Infiltration levels of various immunocytes, such as activated B cell, immature B cell, CD8 T cell, effector memory CD8 T cell, dendritic cell, central memory CD4 T cell, eosinophil, immature dendritic cell, macrophage, MDSCs, natural killer T cell, natural killer cell, regulatory T cell, type 1 T helper cell, and type 17 T helper cell. To further confirm the correlation between the risk signature and immune status, we used the ESTIMATE algorithm to obtain the immune/stromal/estimate scores and tumor purity in the TME and collected survival data to explore the effect of these scores on survival rates. The results revealed that high immune/stromal/estimate scores were associated with prolonged survival in LUAD patients, which is consistent with the result from the previous study (Xiang et al. 2021). The current immune checkpoint biomarkers have limited sensitivity and specificity, meaning that not all patients who are positive for the biomarker will respond to immunotherapy, and some patients who are negative for the biomarker may still respond. Our signature exhibited higher predictive power for the survival status of LUAD patients and response outcomes for immunotherapy and is a potential immune-oncogenic biomarker for prognosis, therapeutic drug selection, and follow-up.

In addition, through drug sensitivity prediction, we screened drugs with better sensitivity for patients in the high-risk scoring group from numerous clinical and preclinical chemotherapy and targeted drugs, such as the current clinical drugs lapatinib (a dual tyrosine kinase inhibitor that inhibits both *EGFR* and *HER2*), docetaxel, and paclitaxel, as well as candidate anti-cancer drugs BI-2536 (a known potent human polo-like kinase 1 inhibitor) and sepantronium bromide (a small molecule survivin inhibitor), and ULK1 (a ULK1 inhibitor). This result provides a basis for the development of new drugs and the selection of clinical medication and is expected to improve the poor prognosis of high-risk patients. Our signature suggests that high-risk patients may derive benefits from EGFR- tyrosine kinase inhibitors (TKIs) and ALK-TKIs, indicating a potential association between our risk scores and EGFR mutations or ALK fusions, which is an important. However, due to the limited proportion of samples with EGFR and ALK alterations in our study cohort, and the lack of clarity regarding the treatments used, a larger sample size is needed to explore the relationship between EGFR/ALK mutations and our risk model.

Above all, we used the data from the public database to identify five immune-related eRNA-driven genes, established a risk prediction model, and verified the signature in multiple datasets. The established signature can be used to predict the survival with high robustness and specificity and assist clinicians to make more beneficial decisions in LUAD. Moreover, specific eRNAs and target genes could be potential therapeutic targets for refractory tumors other than LUAD. In addition, our study provides a potential new predictive biomarker for the prognosis and survival of patients with LUAD and is expected to provide some possible options for improving immunotherapy. However, the effectiveness of risk score signatures needs to be further tested in a larger cohort of LUAD patients. More in vitro and in vivo experiments are needed to validate the biological functions and mechanisms of the genes related to the risk model in LUAD, which will help to better understand the role of these genes in disease development. The interactions between the genes in the risk model and other biological pathways and regulatory factors need further study to reveal more potential mechanisms of LUAD development and prognosis and provide more therapeutic targets. Furthermore, an LUAD cohort with a large sample size is required to further validate and confirm the predictive ability and reliability of our risk model in different clinical situations and independent cohorts.

## Supplementary Information

Below is the link to the electronic supplementary material.

Supplementary file1 (XLSX 34 KB)

Supplementary file2 (XLSX 9 KB)

Supplementary file3 (XLSX 16 KB)

Supplementary file4 Supplementary Figure 1 The respective role of five signature genes on survival. (A) SHC1, (B) GDF10, (C) CCL14, (D) FYN, and (E) NOD1 (TIF 10574 KB)

## Data Availability

The datasets generated during and/or analyzed during the current study are available from the corresponding author on reasonable request.
