# Supplementary figures and images for: Prognostic and onco-immunological value of immune-related eRNAs-driven genes in lung adenocarcinoma
Source: J Cancer Res Clin Oncol. 2024 Apr 11;150(4):188. doi: 10.1007/s00432-024-05687-5 (PMC11008071; doi:10.1007/s00432-024-05687-5)

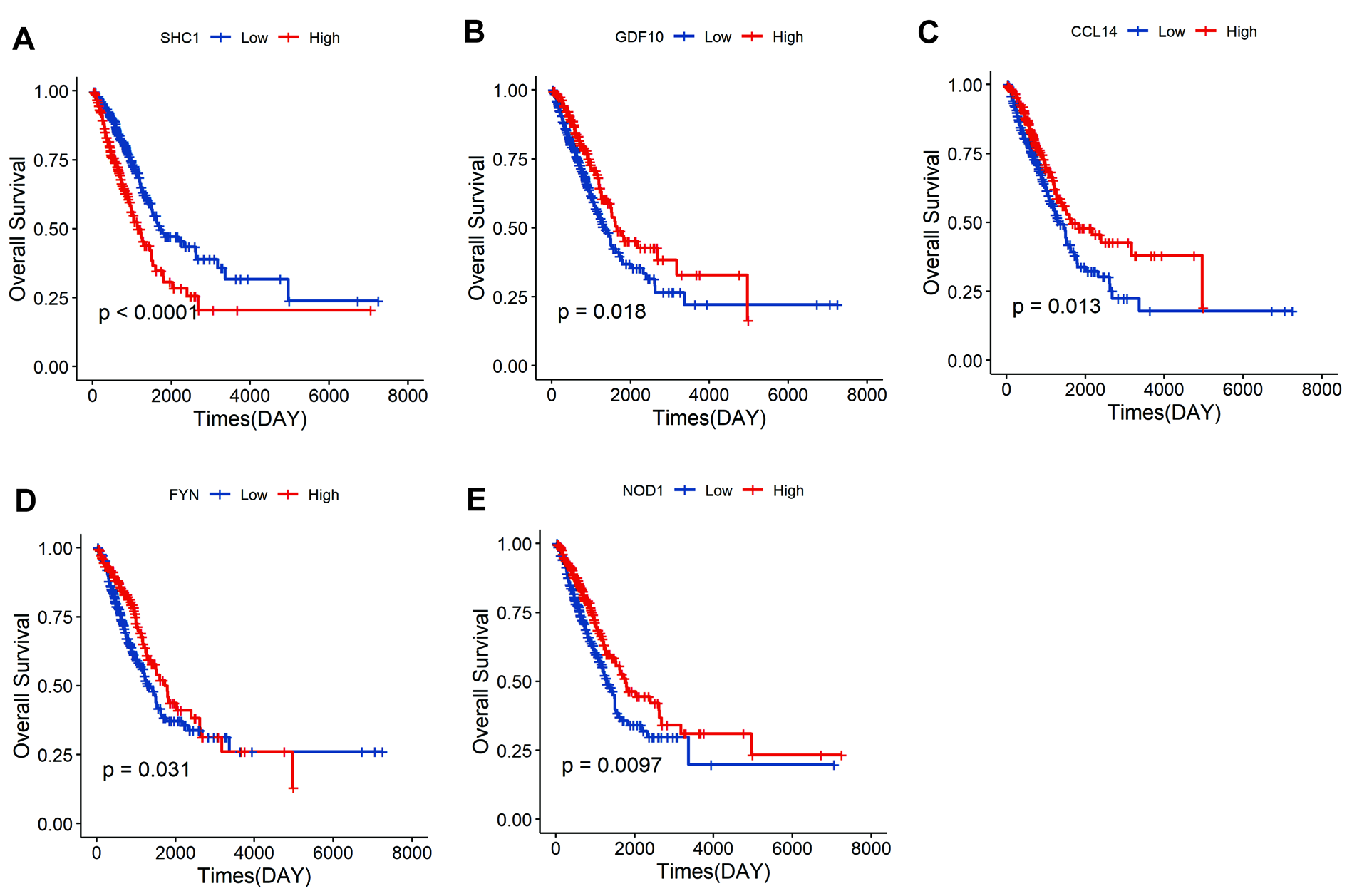

Supplement: Supplementary file 4 — Supplementary file4 Supplementary Figure 1 The respective role of five signature genes on survival. (A) SHC1, (B) GDF10, (C) CCL14, (D) FYN, and (E) NOD1 (TIF 10574 KB) [file 432_2024_5687_MOESM4_ESM.tif]
